# Bevacizumab plus erlotinib versus erlotinib alone for advanced EGFR-mutant non-small cell lung cancer: a meta-analysis of randomized clinical trials

**DOI:** 10.1186/s40001-023-01272-7

**Published:** 2023-08-27

**Authors:** Ruijian Li, Weiyi Li, Fang Zhang, Shanshan Li

**Affiliations:** 1 Department of Radiation Oncology, Yantai Affiliated Hospital of Binzhou Medical University, No 717, Jinbu Road, Yantai, 264000 Shandong China; 2 Department of Respiratory, Yantai Affiliated Hospital of Binzhou Medical University, Yantai, 264000 China

**Keywords:** Bevacizumab, Erlotinib, Non-small cell lung cancer, Subgroup, Meta-analysis

## Abstract

**Objective:**

Previous studies showed that the combination of bevacizumab and erlotinib (combination therapy) significantly prolonged progression-free survival (PFS) but no overall survival (OS) compared to erlotinib alone (monotherapy) for advanced EGFR-mutant non-small cell lung cancer (NSCLC). Two phase III randomized controlled trials (RCTs) had reported the OS results in 2021. This meta-analysis aimed to include the results of the two RCTs to make a decision.

**Materials and methods:**

We systematically searched relevant databases for RCTs on the use of bevacizumab plus erlotinib in advanced EGFR-mutant NSCLC. The main outcomes of interest were PFS, OS, and the reported hazard ratio (HR). Fixed-effect model was used to estimate pooled HR.

**Results:**

Total 5 RCTs with 935 patients were eligible for this meta-analysis. All studies reached their primary study endpoints including PFS and OS. Compared to monotherapy, combination therapy remarkably prolonged PFS (HR = 0.60, 95% confidence interval CI 0.51–0.70; *p* < 0.00001); however, OS was similar between the two groups (HR = 0.90, 95% CI 0.76–1.08; *p* = 0.26). Subgroup analysis demonstrated that in deletion within exon 19 (19del) mutation subgroup, the combination therapy could only prolong PFS (HR = 0.60, 95% CI 0.47–0.76; *p* < 0.0001) but not OS (HR = 1.00, 95% CI 0.73–1.37; *p* = 1.00), and also in leucine-to-arginine substitution in exon 21 (L858R) mutation subgroup (HR = 0.59, *p* < 0.0001 and HR = 0.80, *p* = 0.18, respectively). For patients with brain metastasis at baseline, the combination therapy achieved a significant better PFS than the monotherapy (HR = 0.60, 95% CI 0.39–0.90; *p* = 0.01), and a better OS with the difference marginally significant (HR = 0.69, 95% CI 0.46–1.02; *p* = 0.06).

**Conclusions:**

Combination of bevacizumab and erlotinib can prolong progression-free survival but not overall survival compared to erlotinib alone in advanced EGFR-mutant non-small cell lung cancer patients. The combination therapy not only can prolong progression-free survival but also has a tendency to prolong overall survival for patients with brain metastasis at baseline.

## Introduction

Epidermal growth factor receptor (EGFR) mutation represents the most frequent driver-gene alteration in non-small cell lung cancer (NSCLC), and 90% of EGFR mutations involving a deletion within exon 19 (19del) or a leucine-to-arginine substitution in exon 21 (L858R) [1, 2]. Erlotinib, a small-molecule EGFR tyrosine kinase inhibitor (TKI), is recommended as a first-line therapy for patients with advanced NSCLC harboring the two common EGFR mutations [3]. However, most NSCLC patients treated with erlotinib develop therapeutic resistance, with the median progression-free survival (PFS) of 9.7–13.1 months [4, 5]. To overcome this acquired resistance and improve the PFS, several studies [6–10] have investigated the combination of bevacizumab and erlotinib for the NSCLC patients with EGFR mutations. Most of these studies (ARTEMIS [9], NEJ026 [8], JO25567 [7], BEVERLY [10]) have displayed better PFS in the combination therapy than erlotinib monotherapy, but no overall survival (OS) benefit in the combination therapy. Whether this combination therapy was more effective than monotherapy? Some meta-analyses tried to answer this question. For example, Chen’s meta-analysis [11] and Landre’s meta-analysis [12] both showed that the combination therapy significantly prolonged PFS but no OS compared to monotherapy. However, the conclusions of these meta-analyses were controversial, because the OS results of both ATEMIS study and NEJ026 study were immature at that time. As the OS results of the two studies were published in 2021, we conducted a meta-analysis to provide more reliable and stable evidence to determine the PFS and OS benefit in the combination therapy for advanced EGFR-mutant NSCLC. A secondary objective was to investigate whether efficacy differed between 19del and L858R mutation or between with brain metastasis and without brain metastasis.

## Materials and methods

### Study design

This meta-analysis was conducted according to the Preferred Reporting Items for Systematic Reviews and Meta-Analysis (PRISMA) guidelines.

### Search strategy

The PubMed, EMBASE, and Cochrane Trials databases were rigorously reviewed for randomized controlled trial (RCT) up to June 2022 focusing on the combination of erlotinib and bevacizumab in NSCLC. The complete search terms included: (Carcinoma, Non-Small-Cell Lung [MeSH term] OR Carcinoma, Non Small Cell Lung [Text Word]) AND (Erlotinib Hydrochloride [MeSH term] OR Hydrochloride, Erlotinib [Text Word]) AND (Bevacizumab [MeSH term] OR Avastin [Text Word]). We also manually searched the reference lists for further eligible articles, and the corresponding abstracts at the annual American Society of Clinical Oncology (ASCO) and European Society of Medical Oncology (ESMO) meetings.

### Inclusion criteria

To ensure the quality and reliability of this meta-analysis, only high-quality RCTs that met the following criteria were included: (1) Population: patients with histologically or cytologically confirmed advanced EGFR-mutant NSCLC. (2) Intervention: erlotinib plus bevacizumab. (3) Comparison: erlotinib as a single agent. (4) Outcome: OS, PFS, objective response rate (ORR), and adverse events (AEs).

### Data extraction

Data extraction was performed by two reviewers independently, and disagreement over eligibility of a study was resolved by consensus. The reviewers extracted the key information as following: first author’s name, year of publication, clinical trial information, study design, intervention details, number of patients, pathologic features, EGFR mutation status, and relevant outcomes. Considering that some results (ORR and AEs) remain unchanged until now and they have been proved in previous meta-analyses [11–13] in 2020, the main outcomes of interest in this meta-analysis were PFS, OS, and the available reported hazard ratio (HR) for the outcomes.

### Quality assessment of included studies

The Cochrane Collaboration’s risk of bias tool [14] was adopted to assess risk of bias for each RCT by two investigators. Seven items were used to evaluate heterogeneity in each trial: random sequence generation, allocation concealment, blinding of participants and personnel, blinding of outcome assessment, incomplete outcome data, selective reporting, and other biases. The quality of each RCT was categorized as high, low, or unclear. Discrepancies were resolved by discussion with a third investigator.

### Statistical analysis

Review Manager (version 5.3, the Cochrane Collaboration) was used for data analysis. For the time-to-event variables, the HR and 95% confidence interval CI were extracted from the original RCT. Heterogeneity across studies was assessed with a forest plot and the inconsistency statistic (*I*^2^). If the heterogeneity was moderate or severe (*I*^2^ ≥ 50%), a random-effect model would be applied; otherwise, the fixed-effect model would be applied. Subgroup analysis was conducted according to EGFR mutation subtype (19del and L858R) and brain metastasis at inclusion (with and without brain metastasis). Results were reported with HR and 95% CI for PFS and OS. A two-sided *p* < 0.05 was considered statistically significant. Graphical funnel plot was generated to visually inspect for publication bias.

## Results

### Search results

**Fig. 1:**
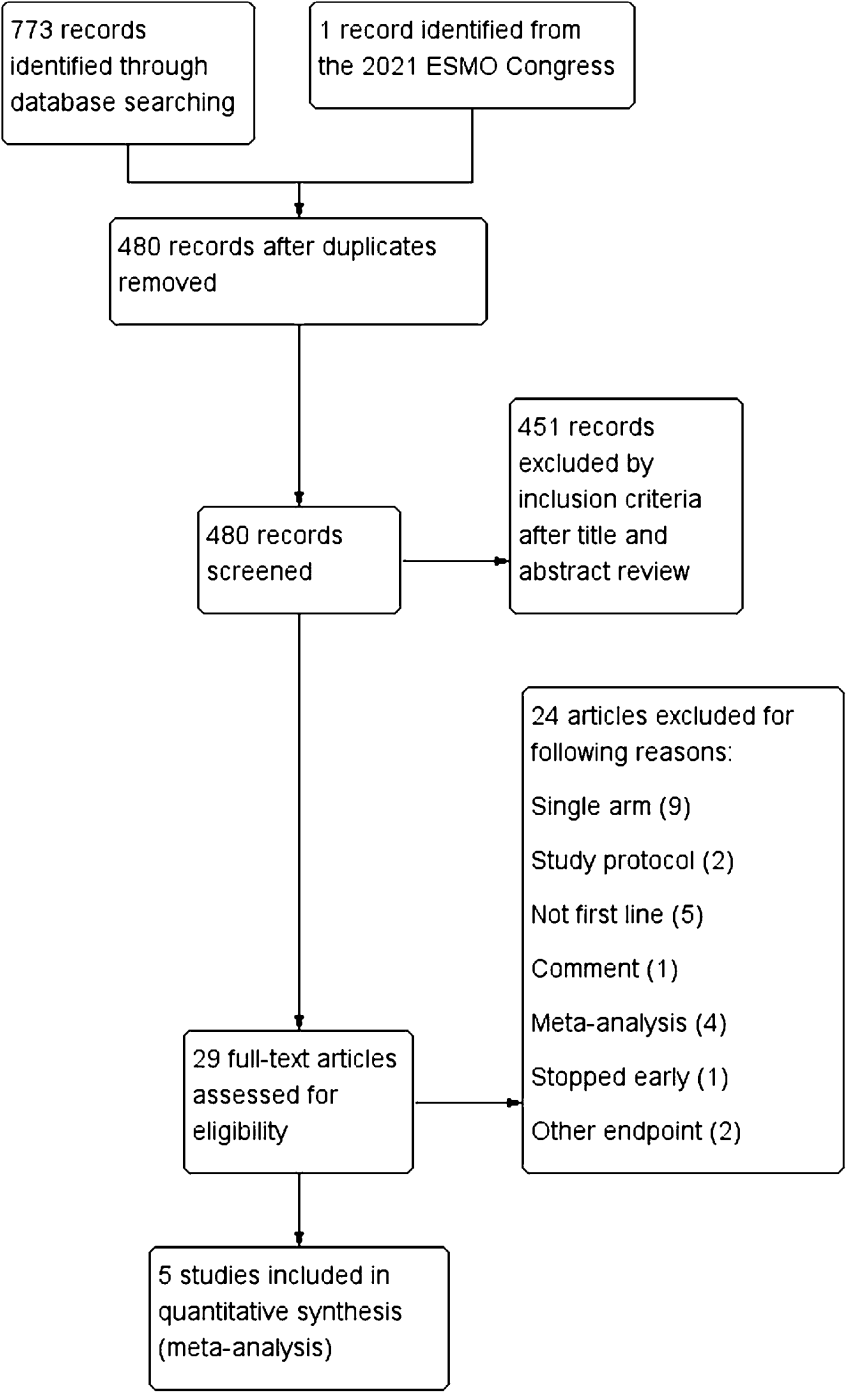
Selection and evaluation process of the eligible studies

A total of 773 eligible studies were identified by searching the databases. The data from the BEVERLY study presented at the 2021 ESMO Congress were also included online. There were 294 duplicate records were removed and the remaining 480 studies were reviewed for title and abstract. Subsequently, 29 potentially eligible studies were assessed by full-text review. Finally, 5 RCTs were selected according to the inclusion criteria (Fig. 1).

### Characteristics and quality assessment of included studies

The characteristics of these 5 RCTs (3 phase III trials and 2 phase II trials) are shown in Table 1, and all the RCTs were judged to have a low risk of bias using the Cochrane Collaboration criteria. Of the 5 RCTs, 3 were performed in Asia, 1 in Italy, and 1 in the United States. A total of 935 patients were enrolled, 553 (59.1%) were women, 467 (49.9%) received combination therapy, 499 were 19del mutation and 430 were L858R mutation. The combination therapy was the erlotinib (150 mg/d) plus bevacizumab (15 mg/kg, Q3w), whereas the monotherapy was administered erlotinib alone.

**Table 1:** Characteristics of the included studies

Study	Country	Phase	EGFR 19del/L858R mutation (no.)	Treatment	Sample size (no.)	PFS (months)	OS (months)
Stinchcombe 2019	US	II	59/29	Combination therapy Monotherapy	43 45	17.9 13.5	32.4 50.6
JO25567 2020	Japan	II	80/72	Combination therapy Monotherapy	75 77	16.4 9.8	47.0 47.4
NEJ026 2021	Japan	III	111/113	Combination therapy Monotherapy	112 112	16.9 13.3	50.7 46.2
CTONG1509 2021	China	III	161/150	Combination therapy Monotherapy	157 154	17.9 11.2	36.2 31.6
BEVERLY 2021	Italy	III	88/66	Combination therapy Monotherapy	80 80	15.4 9.6	33.3 22.8

### Main outcomes analysis

**Fig. 2:**
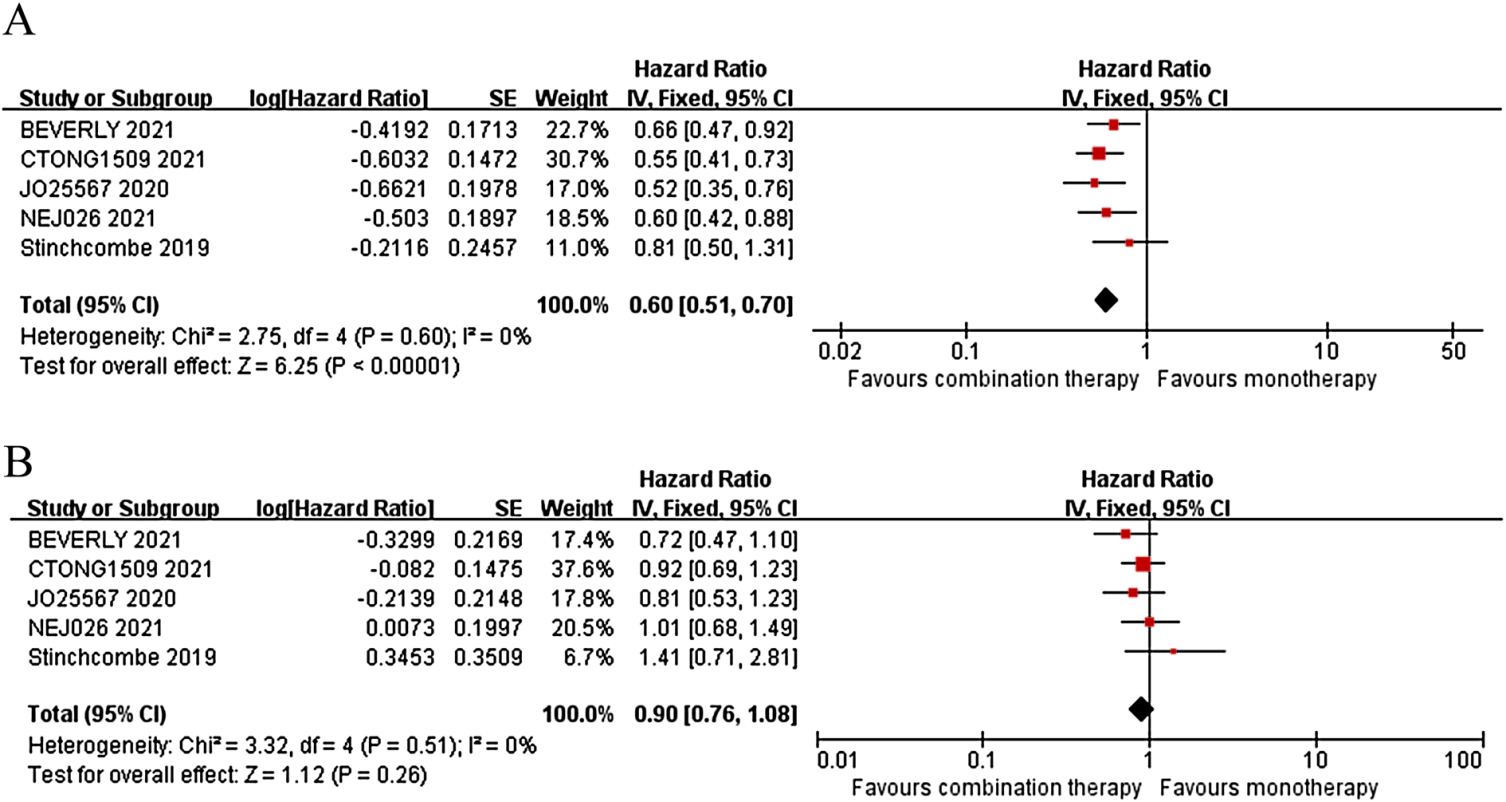
Forest plots of PFS (**A**) and OS (**B**) between combination therapy and monotherapy in all patient population

The combination therapy achieved a significant PFS benefit (HR = 0.60, 95% CI 0.51–0.70; *p* < 0.00001) compared to monotherapy, but failed to be translated into OS benefit (HR = 0.90, 95% CI 0.76–1.08; *p* = 0.26) in all patient population (Fig. 2).

**Fig. 3:**
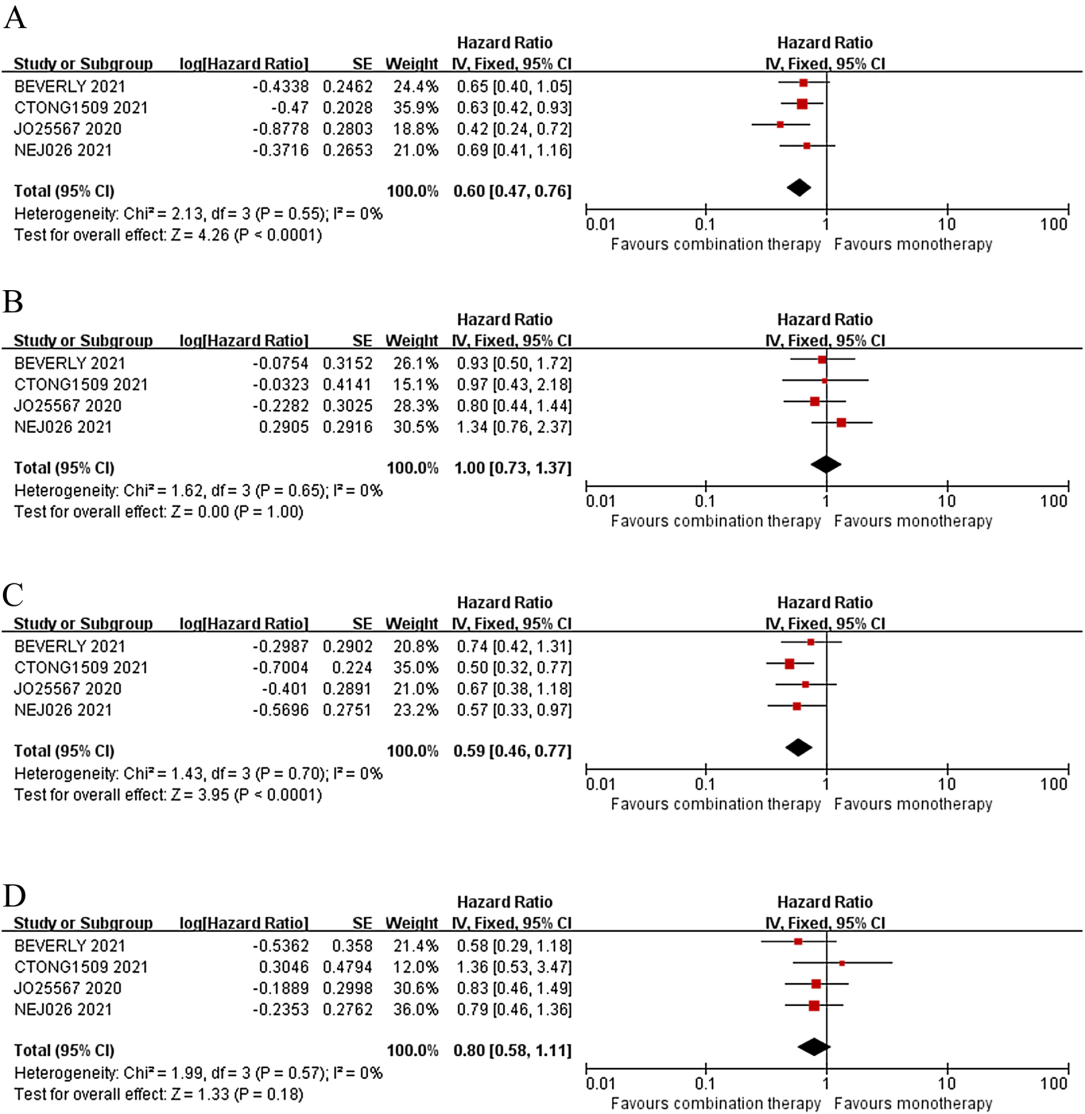
In 19del mutation subgroup, forest plots of PFS (**A**) and OS (**B**) between combination therapy and monotherapy. In L858R mutation subgroup, plots of PFS (**C**) and OS (**D**) between combination therapy and monotherapy

**Fig. 4:**
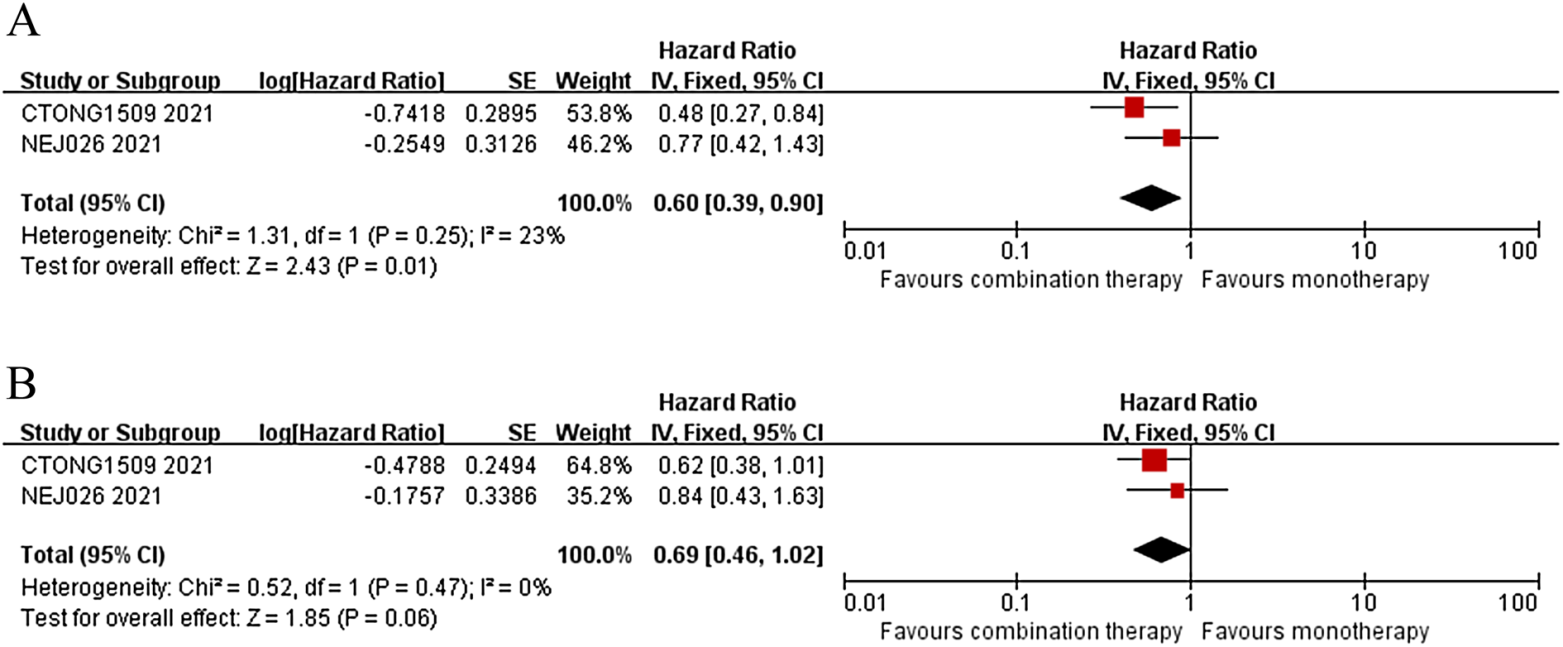
For patients with brain metastasis, forest plots of PFS (**A**) and OS (**B**) between combination therapy and monotherapy

Subgroup analyses stratified by the EGFR mutation subtype and brain metastasis at inclusion were conducted. The PFS of both 19del mutation subtype (HR = 0.60, *p* < 0.0001) and L858R mutation subtype (HR = 0.59, *p* < 0.0001) seemed to be improved by the combination therapy, but OS of the two subtypes were still not prolonged (HR = 1.00, *p* = 1.00 and HR = 0.80, *p* = 0.18, respectively) (Fig. 3). For patients with brain metastasis at inclusion, the combination therapy achieved a significant better PFS than the monotherapy (HR = 0.60, 95% CI 0.39–0.90; *p* = 0.01), and a better OS with the difference marginally significant (HR = 0.69, 95% CI 0.46–1.02; *p* = 0.06) (Fig. 4).

## Discussion

Erlotinib, a first-generation reversible TKI, targets the EGFR signaling pathway. Bevacizumab, an anti-vascular endothelial growth factor (VEGF) monoclonal antibody, targets the VEGF signaling pathway. Additional clinical benefits may be conferred by the dual blockade of the EGFR and VEGF pathways that critical to tumor growth, metastasis and angiogenesis [15, 16]. Therefore, quite a few clinical studies [6–10] had attempted this combination therapy: erlotinib plus bevacizumab. Although some clinical studies had achieved prolongation of PFS, there was no corresponding prolongation of OS. This meta-analysis is motivated by uncertainty about the value of combination therapy compared with monotherapy.

According to the results of this meta-analysis, the combination therapy significantly prolonged PFS (Fig. 2A), but did not prolong OS (Fig. 2B) compared to monotherapy for advanced EGFR-mutant NSCLC patients. Although final survival outcomes from study ATEMIS and NEJ026 were included in this meta-analysis, there was still no prolongation of OS, consistent with the findings of three previous meta-analyses [11–13]. Based on the advantages of dual blockade of EGFR and VEGF pathways, the prolongation of PFS by combination therapy is currently indisputable, while there are other relevant reasons for the failure to prolong OS. For instance, OS might have been influenced by the subsequent treatments used after disease progression, especially the use of third-generation TKI osimertinib. In the study ATEMIS, more patients from the monotherapy group received osimertinib as subsequent treatment than in the combination therapy group (29.2% vs 17.2%), and this ratio was 28.9% vs 23.3% in another study [6]. In addition, the number of patients receiving subsequent anticancer treatment was also greater in the monotherapy group in the ATEMIS study (50.0% vs 33.8%). Thirdly, Grade ≥ 3 adverse events occurred more frequently in the combination therapy group than in the monotherapy group [6, 9]. Therefore, the combination therapy might be compromised because of the above problems.

In general, the L858R mutation subgroup derives less benefit from first-line EGFR TKIs than the 19del mutation subgroup, which has been repeatedly demonstrated in previous clinical trials and real-world studies [17–19]. The prognosis of patients with L858R mutation urgently needs to be improved in the era of TKI targeted therapy [20]. Would adding a VEGF inhibitor in combination with the TKI change the subgroup’s outcome? Therefore, subgroup analysis was conducted in this meta-analysis. In 19del mutation subgroup, the HR for PFS was 0.60 with a 95% confidence interval of 0.47 to 0.76 (*p* < 0.0001), and the HR for OS was 1.00 with a 95% confidence interval of 0.73 to 1.37 (*p* = 1.00) (Fig. 3A, B). In L858R mutation subgroup, the HR for PFS was 0.59 with a 95% confidence interval of 0.46 to 0.77 (*p* < 0.0001), and the HR for OS was 0.80 with a 95% confidence interval of 0.58 to 1.11 (*p* = 0.18) (Fig. 3C, D). The subgroup analysis showed that the combination therapy only prolonged PFS, but not OS, regardless of L858R or 19del mutation, which was consistent with the results of overall population. When comparing the HR value alone, both PFS and OS of L858R mutation were improved more significantly than those with 19del mutation. This suggests that the combination therapy can reverse the poor prognosis of L858R mutation subgroup, and the combination therapy may be one of the treatment options for patients with L858R mutation [21, 22].

Presence of brain metastasis is a well-known adverse prognostic factor and common site of disease progression in EGFR-mutant NSCLC. Previous study showed that the anti-VEGF drug bevacizumab was effective in treating brain metastasis [23], and also could significantly reduce the incidence of brain metastasis [24]. In two retrospective studies [25, 26], bevacizumab combined with TKI prolonged the OS in patients with brain metastasis compared to TKI monotherapy. Of all the 5 RCT studies, only 2 studies [8, 9] conducted further subgroup analysis for patients with brain metastasis, and thus only the 2 studies were included. The subgroup analysis suggested that the combination therapy not only provided significant improvement in PFS (HR = 0.60, 95% CI 0.39–0.90; *p* = 0.01), but also had a positive trend to prolong OS (HR = 0.69, 95% CI: 0.46–1.02; *p* = 0.06) in patients with brain metastasis (Fig. 4A, B). The statistical *P*-value did not reach significant significance, which may be explained by the following reasons. First of all, only two RCTs were included. Secondly, the treatment-related adverse effects associated with the combination therapy [6, 8, 9]. However, it had given us good enlightenment and pointed out the direction of future research. It is the only way to include more large-scale RCT studies.

It is worth mentioning that osimertinib has replaced erlotinib as the preferred first-line treatment for advanced EGFR-mutant NSCLC patients and second-line treatment for patients with EGFR T790M mutation. Can osimertinib combined with bevacizumab further prolong PFS and OS in patients with EGFR-mutant NSCLC? Several studies had presented the results. BOOSTER [27] and WJOG8715L [28] were two studies of second-line treatment, and neither study showed an extension of PFS and OS. WJOG9717L [29] was a study of first-line treatment, and it also showed that PFS was not prolonged in the combination group, and the OS data were immature. Despite only three phase II studies, the prospects for combination therapy are not promising.

## Conclusion

This meta-analysis reveals that the combination of erlotinib and bevacizumab can prolong progression-free survival but not overall survival compared to erlotinib alone in advanced EGFR-mutant non-small cell lung cancer. For patients with brain metastasis, the combination therapy not only can prolong progression-free survival, but also has a tendency to prolong overall survival. This combination therapy model deserves to be considered in the future, especially for patients with brain metastasis.

## Data Availability

All data generated or analyzed during this study are included in the published RCT articles (and its supplementary information files).
